# Expression of Pluripotency and Oocyte-Related Genes in Single Putative Stem Cells from Human Adult Ovarian Surface Epithelium Cultured *In Vitro* in the Presence of Follicular Fluid

**DOI:** 10.1155/2013/861460

**Published:** 2013-02-28

**Authors:** Irma Virant-Klun, Thomas Skutella, Mikael Kubista, Andrej Vogler, Jasna Sinkovec, Helena Meden-Vrtovec

**Affiliations:** ^1^Department of Obstetrics and Gynecology, University Medical Centre Ljubljana, Slajmerjeva 3, 1000 Ljubljana, Slovenia; ^2^Institute for Anatomy and Cell Biology, Medical Faculty, University of Heidelberg, Im Neuenheimer Feld 307, 69120 Heidelberg, Germany; ^3^TATAA Biocenter AB, Odinsgatan 28, 41103 Göteborg, Sweden; ^4^Institute of Biotechnology AS CR, Lb Building, 14220 Prague, Czech Republic

## Abstract

The aim of this study was to trigger the expression of genes related to oocytes in putative ovarian stem cells scraped from the ovarian surface epithelium of women with premature ovarian failure and cultured *in vitro* in the presence of follicular fluid, rich in substances for oocyte growth and maturation. Ovarian surface epithelium was scraped and cell cultures were set up by scrapings in five women with nonfunctional ovaries and with no naturally present mature follicles or oocytes. In the presence of donated follicular fluid putative stem cells grew and developed into primitive oocyte-like cells. A detailed single-cell gene expression profiling was performed to elucidate their genetic status in comparison to human embryonic stem cells, oocytes, and somatic fibroblasts. The ovarian cell cultures depleted/converted reproductive hormones from the culture medium. Estradiol alone or together with other substances may be involved in development of these primitive oocyte-like cells. The majority of primitive oocyte-like cells was mononuclear and expressed several genes related to pluripotency and oocytes, including genes related to meiosis, although they did not express some important oocyte-specific genes. Our work reveals the presence of putative stem cells in the ovarian surface epithelium of women with premature ovarian failure.

## 1. Introduction

From the literature it is known that oocyte-like cells expressing different oocyte-specific genes can be developed *in vitro* from mouse embryonic stem cells (mESCs) [1–8], human embryonic stem cells (hESCs) or human induced pluripotent stem cells (hiPSCs) [9–11], stem cells from human amniotic fluid [12], from porcine fetal skin [13, 14], and even from rat pancreatic stem cells [15].

Although *in vitro* oogenesis from animal and human ESCs could represent a model to study the mechanisms of oogenesis and their pathologies, the potential oogenesis from the autologous ovarian stem cells would be of great advantage because it may be realistically applied in human medicine in the future. Ovarian stem cells may play an important role. More studies have already confirmed the presence of pluripotent/multipotent stem cells in neonatal and adult ovaries of mice [6, 16, 17] and proposed human ovarian surface epithelium (OSE) as an important source of stem cells in human [18–22] and other mammalian species, such as sheep and monkey [22]. Moreover, White et al. have recently published the existence of rare mitotically active cells—germline stem cells—with a gene expression profile that is consistent with primitive germ cells, which can be purified from adult human ovarian cortical tissue by fluorescence-activated cell sorting-based protocol [23]. They have proven that these cells can be expanded for months *in vitro* and can spontaneously be developed into haploid oocyte-like cells with diameters of up to 35–50 *μ*m. When marked with green fluorescence protein (GFP) and transplanted into human ovarian cortical biopsies, the follicles containing GFP-positive oocytes were formed 1-2 weeks after the xenotransplantation into immunodeficient female mice. They concluded that the ovaries of reproductive-age women possess rare mitotically active germ cells that can be propagated *in vitro* and can generate oocytes *in vitro* and *in vivo*. 

Premature ovarian failure (POF) is one of the most serious indications of female infertility resulting in nonfunctional ovaries without mature follicles and oocytes before the age of 40 years [24]. It is characterized by high levels of gonadotropins in the blood and amenorrhea. These women have no mature oocytes and do not conceive and cannot bear their own child. Any potential to regenerate the nonfunctional ovaries in these women would be of the greatest importance in reproductive medicine.

The aim of this study was to culture *in vitro* putative stem cells from the OSE of nonfunctional ovaries in the presence of donated follicular fluid, rich in substances important for oocyte growth and maturation to trigger their growth and the expression of genes related to human oocytes. Because the genetic status of oocyte-like cells developed *in vitro* from stem cells is still poorly understood, these cells were analyzed by detailed single-cell gene expression profiling in comparison to human embryonic stem cells, oocytes at different stages of maturity, and somatic fibroblasts to elucidate their genetic status. In this way we made some steps further from our previous work. The primitive oocyte-like cells developed in this study expressed several genes characteristic of pluripotent stem cells and oocytes, including some genes related to meiosis, but were more “stem cells” than “oocytes” at this stage. 

## 2. Materials and Methods

In five women with premature ovarian failure (POF) and with no naturally present mature follicles or oocytes the putative ovarian stem cells were retrieved by OSE brushing. The mean female age was 34 years (range: 21–39 years). Each woman donated a part of her ovarian tissue for the purpose of research after having the study explained in detail and then provided written consent to participate. All women were characterized by irregularities in their menstrual cycle, elevated levels of gonadotropins (follicle-stimulating hormone (FSH) and luteinizing hormone (LH)) in their blood serum, and a thin endometrium, as can be seen in Table 1. The molecular status of oocyte-like cells developed *in vitro* was compared to hESCs (H1 cell line, WiCell Research Institute, Madison, WI, USA) and nonfertilized oocytes from the *in vitro* fertilization programme, donated for the purpose of research with the written consents of the donating women. There was no financial recompense to the donors of oocytes. This research was approved by the Slovenian Medical Ethical Committee (Ministry of Health of the Republic of Slovenia, No. 110/10/05).

### 2.1. Ovarian Stem Cell Retrieval

In each of the five women with POF the putative ovarian stem cells were retrieved by soft OSE brushing of the whole ovary and small ovarian cortex biopsy (approximately 0.3 mm^3^), retrieved by the usual diagnostic laparoscopic procedure. A part of the ovarian cortex biopsy was sent to the histopathological service lab to evaluate the presence of follicles or oocytes after haematoxylin-eosin (HE) staining, common in everyday clinical practice; another part of the ovarian cortex tissue was used to set up a cell culture. The ovarian cortex biopsy was put in a volume of 2.5 mL of warmed and preincubated supplemented DMEM/F-12 culture medium (composition described below). The OSE layer was mechanically scraped several times using a sterile surgical blade (Swann-Morton, Sheffield, United Kingdom, ref. 0501), which was washed in the surrounding culture medium to retrieve a scraped suspension of cells to set up cell cultures.

### 2.2. Ovarian Surface Epithelium Cell Culture

The OSE cell cultures were set up using OSE brushings of cortex biopsies. In each patient the OSE cell culture was set up by dropping five drops of the cell suspension from brushed OSE into the DMEM/F-12 culture medium, prepared as follows: Dulbecco's Modified Eagle's Medium (DMEM)/Nutrient Mixture F-12 Ham with L-glutamine, phenol red, and 15 mM HEPES (Sigma Aldrich), supplemented with 3.7 g/L NaHCO_3_, 1% penicillin/streptomycin, and 0.5% gentamycin, and with the pH adjusted to 7.4 with 1 M NaOH; this culture medium was supplemented with 20% (v/v) donated follicular fluid from the *in vitro* fertilization programme. In each patient the cell culture was set up in 12 IVF Multidish Four well Nunclon dishes (Nunc, Roskilde, Denmark, ref. 144444). Each well was filled with 350 *μ*L of preincubated culture medium to which drops of the cell suspension from brushed OSE were added. The cells were cultured for two months in a CO_2_ incubator (Heraeus 6000, Heraeus Holding, Hanau, Germany) at 37°C and 6% CO_2_ in the air and monitored daily under a heat-staged inverted microscope (ECLIPSE 2000-S, Nikon, Tokyo, Japan) equipped with a Digital Sight camera (Nikon, Tokyo, Japan). 

### 2.3. Preparation of Follicular Fluid

The follicular fluid, retrieved after the written consent of the young donor who had normal ovarian function, was used immediately after the removal of the oocytes (to be fertilized *in vitro*) in order not to coagulate. It was centrifuged for 10 minutes at 2,500 rpm (349 ×g). The supernatant was filtered through a sterile Sartorius Minisart 0.45 *μ*m filter to remove all the cells. The filtered supernatant was heat inactivated at 56°C for 45 minutes. Then it was aliquoted and stored at −20°C until its use. When it was used, an aliquot of prepared follicular fluid was thawed at the room temperature and added to the culture medium. This fluid contained substances important for oocyte growth and differentiation and was added to provide an ovarian-like growth milieu.

### 2.4. Analyses of Reproductive Hormones in Culture Medium

In the samples of culture medium with added follicular fluid which were taken from two different OSE cell cultures after 15–17 days of culturing the concentrations of estradiol, progesterone, androstenedione, and testosterone were measured in comparison with the same medium with added follicular fluid and without cell culture and the same medium without follicular fluid and without cell culture. Estradiol was measured by a chemoluminescent, competitive, immunochemical method by reagents (Immulite, Siemens, USA) which included alkaline phosphatase-conjugated estradiol and highly specific polyclonal rabbit antiestradiol antibodies on paramagnetic particles. Progesterone was measured by a chemoluminescent, noncompetitive, immunochemical method by reagents (Immulite, Siemens, USA) which included alkaline phosphatase-conjugated progesterone and highly specific monoclonal rabbit antiprogesterone antibodies on paramagnetic particles. Both hormones were analyzed by Immulite analyzer (Siemens, USA). Androstenedione was measured by a specific double antibody RIA using 125 I-labeled hormones (Diagnostic Systems Laboratories, Webster, TX, USA). Testosterone level was determined by RIA (DiaSorin and DPC, Los Angeles, CA, USA). All procedures were performed according to standardized procedures at the Institute of Nuclear Medicine, University Medical Centre Ljubljana.

### 2.5. Single-Cell Gene Expression Analyses Using the Fluidigm BioMark System

Gene expression analyses of single oocyte-like cells in comparison with single hESCs and groups of five, ten, and twenty hESCs of H1 line (positive controls), single nonfertilized oocytes from the *in vitro* fertilization programme (positive controls), and single human fibroblasts and groups of five, ten, and twenty fibroblasts of F161 line (negative controls) were performed using the BioMark Real-Time quantitative PCR (qPCR) system (Fluidigm, San Francisco, CA, USA). In all oocyte-like cells, mechanically removed from the cell cultures expressions of 56 genes, 21 genes characteristic of pluripotent stem cells (*KIT*, *KIT LIG*, *OCT4A*, *NANOG*, *MYC*, *KLF4*, *SOX-2*, *UTF1*, *TDGF1*, *LIN28B*, *TERT*, *CD9*, *LIN28*, *NANOS*, *CDH1*, *STAT3*, *REX1*, *MEST*, *CRKRS*, *STELLA*, and *GDF3*) and 34 genes typical of oocytes (*VASA*, *DAZL*, *STELLA*, *GFRa1*, *KIT*, *KIT LIG*, *DNMT3B*, *DNMT1*, *BMP15*, *ZP1*, *ZP2*, *ZP3*, *ZP4*, *SCP1*, *SCP2*, *SCP3*, *BUB1*, *BUB3*, *NOBOX*, *MSH5*, *NLRP5*, *FMN2*, *HIFOO*, *MLH1*, *ZAR1*, *REC8*, *PRDM1/BLIMP1*, *FIGLA*, *STAG3*, *DMC1*, *SMC1*, *CD9*, *BNC1*, and *CCNB1*) and of the housekeeping gene *GAPDH* were analyzed. The inventoried TaqMan assays (20x, Applied Biosystems, Life Technologies, Carlsbad, CA, USA) were pooled to a final concentration of 0.2x for each of the 56 assays. The cells to be analyzed were harvested directly into 9 *μ*L RT-PreAmp Master Mix: 5.0 *μ*L CellsDirect 2x Reaction Mix (Invitrogen, Life Technologies, Carlsbad, CA, USA); 2.5 *μ*L 0.2x assay pool; 0.2 *μ*L RT/Taq Superscript III (Invitrogen, Life Technologies); 1.3 *μ*L TE buffer. The harvested cells were immediately frozen and stored at −80°C. Cell lysis and sequence-specific reverse transcription were performed at 50°C for 15 minutes. The reverse transcriptase was inactivated by heating to 95°C for 2 minutes. Subsequently, in the same tube cDNA went through limited sequence-specific amplification by denaturing at 95°C for 15 seconds and then annealing and amplification at 60°C for four minutes for 14 cycles. These preamplified products were diluted 5-fold prior to analysis with the Universal PCR Master Mix and inventoried TaqMan gene expression assays (ABI) in 96.96 Dynamic Arrays on a BioMark System. Each sample was analyzed in two technical replicates. Ct values obtained from the BioMark System were transferred to the GenEx software (MultiD) to analyze the gene expressions. 

### 2.6. Analyses of Single-Cell Gene Expressions by GenEx Software (MultiD)

Ct values were obtained from the BioMark System and transferred to the GenEx software (MultiD Analyses, Göteborg, Sweden). Missing data in the BioMark System were given a Ct of 999. These were removed in GenEx. Also Ct values larger than 25 were removed (cut-off value > 25), since samples with such high Ct values in the BioMark 96 × 96 microfluidic card were expected to be negative, and these readings were unreliable. Technical repeats were then averaged. Missing data were then replaced by the highest Cq + 1 for each gene. This corresponded to assigning a concentration to these samples that was half of the lowest concentration measured and was motivated by sampling ambiguity. There was also a need to handle missing data for downstream classification with multivariate tools. Linear quantities were calculated relative to the sample having lowest expression, and data were converted to log_2_ scale. Because of single-cell gene expression analyses, normalization to the housekeeping genes was not performed. The data were now prepared for multivariate analysis to classify the samples based on the combined expression of all the genes. Heatmap clustering (Ward's Algorithm, Euclidean Distance Measure), hierarchical clustering (Ward's Algorithm, Euclidean Distance Measure), and principal component analysis (PCA) were performed. In addition, descriptive statistics were calculated individually for the genes using a 0.95% confidence level, and groups were compared using 1-way ANOVA (Tukey-Kramer's pairwise comparison) and unpaired 2-tailed *t*-test. According to Dunn-Bonferroni, corrected statistical significance was set at *P* < 0.00244 for genes of pluripotency or *P* < 0.00151 for oocyte-specific genes to account for false positives due to multiple testing.

### 2.7. DAPI Staining of Cell Nuclei

To monitor the cell nuclei, single oocyte-like cells were isolated from the cultures and put into a drop of Vectashield mounting medium for fluorescence with DAPI (Vector Laboratories, Peterborough, United Kingdom) and observed under a fluorescence microscope (ECLIPSE E-600 with a Digital Sight camera, magnifications of 200/400x, Nikon, Tokyo, Japan) after 20 minutes of incubation at the room temperature and in the dark.

## 3. Results

### 3.1. Histopathology of Ovarian Tissue

**Figure 1:**
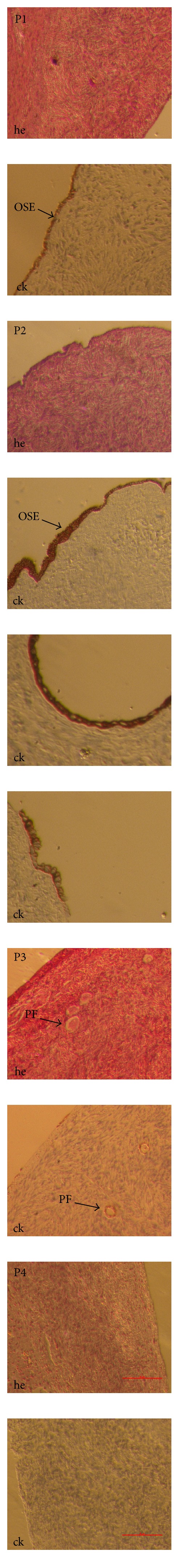
Histology of ovarian sections after haematoxylin-eosin (he) and cytokeratin (ck) stainings in patients (P1, P2, P3, and P4) with POF. It confirmed no naturally present follicles or oocytes in their ovarian cortex, except for primordial follicles in P3. In P5 the histology has been performed in another medical institution. (inverted microscope, Hoffman, magnification 40/100x.) Legend: OSE: ovarian surface epithelium (brown stained), PF: primordial follicle.

A part of each ovarian cortex biopsy was sent to the histopathological routine service. In four of them no follicles or oocytes were observed in their ovarian cortex, and in one biopsy only a few primordial follicles were found after haematoxylin-eosin (HE) staining (Figure 1). Cytokeratin (CK7) staining revealed the presence of OSE in all five ovarian biopsies.

### 3.2. *In Vitro* Culture of Scraped Ovarian Surface Epithelium

**Figure 2:**
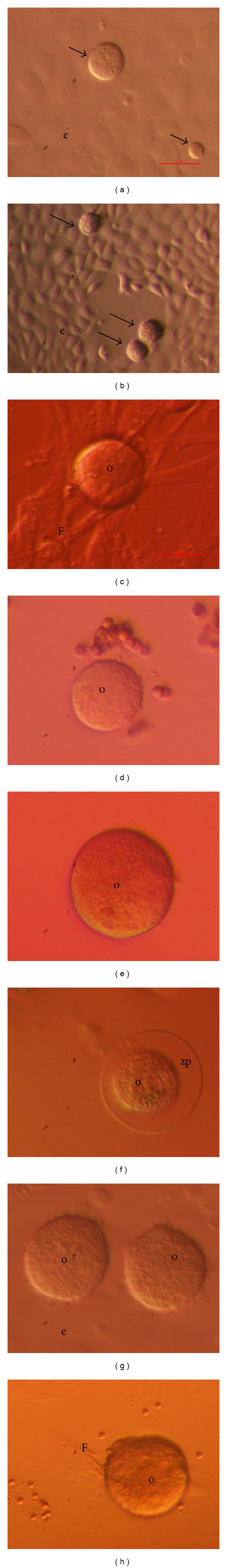
Primitive oocyte-like cells (arrows) developing in ovarian surface epithelium cell cultures set up by ovarian cortex biopsy scrapings: (a, b) among epithelial cells, (c) attached to autologous ovarian fibroblasts, (d, e) oocyte-like cells, (f) growing primitive oocyte-like cell with zona pellucida-like structure, (g) oocyte-like cells, and (h) primitive oocyte-like cell attached to fibroblast. Scale bars: (a, b) 100 *μ*m, (c–h) 50 *μ*m. Legend: e: epithelial cells, F: fibroblast, o: oocyte-like cell, and zp: zona pellucida-like structure.

**Figure 3:**
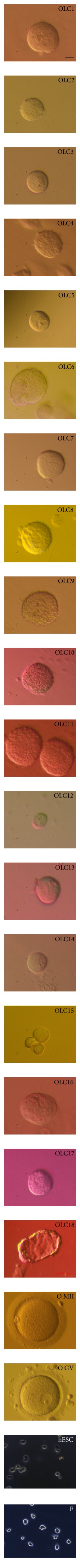
Single cells analyzed on expression of genes: eighteen single primitive oocyte-like cells (OLC1–18), developed *in vitro* (OLC18 further developed in a parthenogenetic blastocyst-like structure) and cultured for up to one month (OLC1–8 and OLC16–18 for 15 days, OLC9–11 for 30 days, and OLC12–15 for 5 days), one mature (O MII) and one immature (O GV) oocyte (inverted microscope, Hoffman, scale bar: 10 *μ*m), and human embryonic stem cells (hESCs) and fibroblasts (F) under a phase-contrast microscope (magnification 200x).

Ovarian fibroblasts were the first cells to attach to the bottom of the dish. They were observed from approximately the second day of culture as elongated cells spreading the dish bottom. After five days, the first large, round cells with different diameters appeared in the culture (Figures 2(a) and 2(b)). Most of them grew attached to the autologous ovarian fibroblasts (Figure 2(c)). The largest cells had diameters of 50–60 *μ*m and had different granularity to their cytoplasm. Some of them had low-granular cytoplasm and resembled primitive oocytes (Figures 2(d) and 2(e)). Henceforth, we refer to them as primitive oocyte-like cells. Rarly they developed zona pellucida-like structures (Figure 2(f)). Some of them developed into parthenogenetic blastocyst-like structures, as can be seen in Figure 3 (OLC18). The primitive oocyte-like cells that developed showed no signs of degeneration during extended cell culturing of up to two months and retained the round shape.

### 3.3. Concentrations of Reproductive Hormones in Culture Medium

All samples of culture medium with added follicular fluid were characterized by very high concentrations of estradiol and progesterone in comparison with reference serum values during different phases of female menstrual cycle (Table 2). They also contained significant concentrations of androstenedione and testosterone in comparison with culture medium without added follicular fluid. In samples of culture medium from ovarian cell cultures the concentrations of all hormones were lower than in samples with added follicular fluid, but without ovarian cell culture; these hormones were depleted/converted by ovarian cell cultures. Also a pure culture medium without added follicular fluid contained a low level of estradiol thus reflecting the presence of phenol red, which is known to express weak estrogenic activity in tissue culture media [25].

### 3.4. Characterization of Individual Primitive Oocyte-Like Cells Differentiated *In Vitro* by Expression Profiling of Pluripotent Stem Cell and Oocyte-Specific Genes

In samples from all patients who had no follicles or oocytes in their ovarian cortex, 18 primitive oocyte-like cells differentiated *in vitro* (Figure 3) and cultured for up to one month (from 5 to 31 days) were mechanically removed from the culture with a glass pipette. Expression of 56 genes was measured in each primitive oocyte-like cell. Twenty-one genes were characteristic of pluripotent stem cells, 34 genes were typical of oocytes, and one gene was housekeeping gene (see Section 2). Nineteen individual nonfertilized oocytes from the *in vitro* fertilization programme collected at different stages of maturation (six germinal vesicle(GV), four metaphase I-MI, four metaphase II-MII, and five *in vitro* matured(IVM) oocytes) and hESCs of the H1 line (three single cells, a group of five cells, a group of ten cells, and a group of twenty cells) were used as positive controls, and human adult fibroblasts from the F161 line (three single cells, a group of five cells, a group of ten cells, and a group of twenty cells) were used as negative controls.

#### 3.4.1. Comparison of Gene Expression Profiles for All Analyzed Genes in All Analyzed Types of Cells

**Figure 4:**
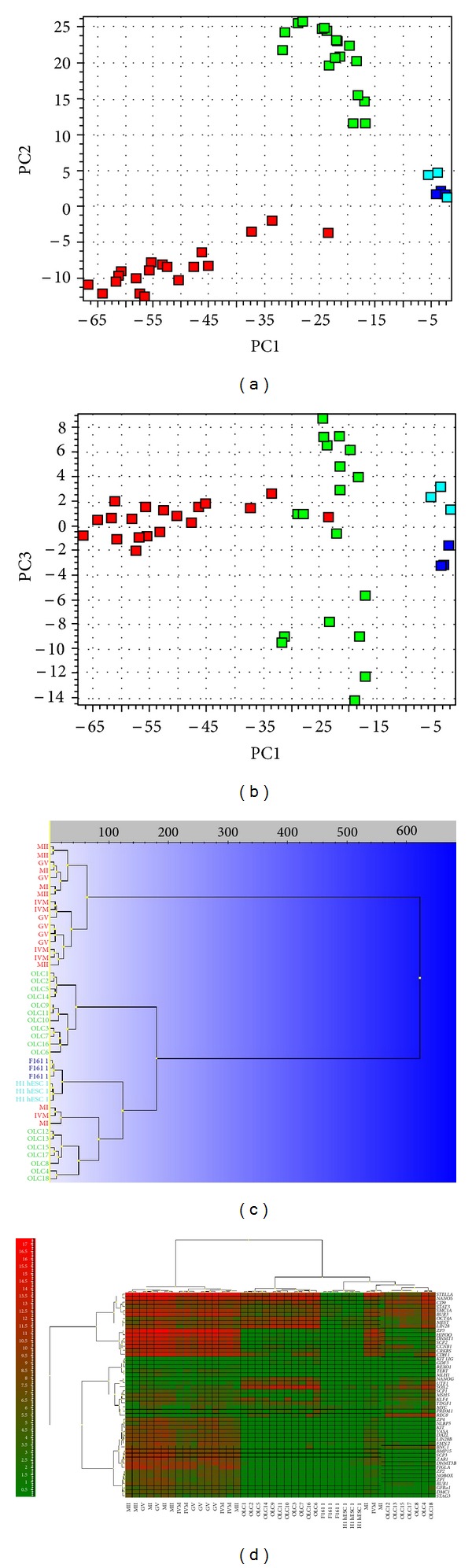
Single-cell gene expression profile of all analyzed genes in primitive oocyte-like cells developed *in vitro* (OLCs). Comparison with human embryonic stem cells (H1 hESCs), nonfertilized oocytes from the *in vitro* fertilization programme (O) and fibroblasts (F161) revealed the expression of several genes related to pluripotent stem cells and oocytes. (a) Principle component analysis (PC1 versus PC2). (b) Principle component analysis (PC2 versus PC3). (c) Hierarchical clustering. (d) Heatmap clustering. Legend of analyzed cells:OLC(1–18): primitive oocyte-like cells developed *in vitro*; O: oocytes; H1 hESC: human embryonic stem cells of line H1 (1: one cell, 5: five cells, 10: ten cells, and 20: twenty cells); F161: fibroblasts of F161 line (1: one cell, 5: five cells, 10: ten cells, 20: twenty cells); MII: mature, metaphase II oocytes; IVM: immature, *in vitro* matured oocytes; MI: immature, metaphase I oocytes; GV: immature, germinal vesicle oocytes from the *in vitro* fertilization programme. Cell group colours: red: oocytes from the *in vitro* fertilization programme, green: primitive oocyte-like cells, developed *in vitro*, aquamarine: hESCs, and dark blue: fibroblasts.

Different types of cells express different genes, and when comparing the expression among the cells we analyze, we expected to see significant differences (Figure 4). There is substantial natural variation in the transcript levels of individual cells of the same kind because of temporal fluctuations caused by transcriptional bursting [26, 27]. The cells of different kinds cannot be unambiguously distinguished based on the expression of any individual gene; it is rather more effective to separate them based on the correlated expression of the panel of genes [28]. The most common and powerful tools to categorize samples based on the expression of multiple genes are the principle component analysis (PCA) and hierarchical clustering [29]. PCA identifies the combinations of genes that account for most of the variation in the measured data and can be used to visually cluster samples based on similar expression profiles in a scatter plot with axes that represent those optimal linear combinations of genes. Those optimum combinations are called principal components (PCs).  Figure 4(a) shows all the analyzed single-cell samples clustered in a PC1 versus PC2 scatter plot.

In this study most of cells were either oocytes or primitive oocyte-like cells, and therefore the mathematical model developed is dominated by features that distinguish between oocytes and oocyte-like cells. As a consequence, the fibroblasts and hESCs do not distinguish themselves appreciably in this comparison. In cases like this, biologically relevant information may be hidden in higher order PCs. The oocyte-like cells, hESCs, and fibroblasts were all found at high PC1 values, which reflects a shared high expression of the genes behind PC1 among them. Along PC3 the fibroblasts and hESCs clearly separated (Figure 4(b)). This suggests that they have different expression among the genes defining PC3. Moreover, primitive oocyte-like cells also separated along PC3 (Figure 4(b)). This suggests there are two kinds of oocyte-like cells that share features either with the hESCs or with the fibroblasts. Eight primitive oocyte-like cells shared features with the fibroblasts and were more “somatic,” possibly, whereas ten primitive oocyte-like cells shared features with the hESCs and were putative stem cells. Primitive oocyte-like cells sharing features with the hESCs also shared features with three oocytes: two immature MI oocytes and one *in vitro* matured(IVM) oocyte, as seen in Figures 4(c) and 4(d).

In hierarchical clustering (heatmaps), sixteen oocytes group together, while three oocytes (two immature MI oocytes and one *in vitro* matured(IVM) oocyte) cluster with seven primitive oocyte-like cells developed *in vitro* (Figure 4(d)). hESCs and fibroblasts cluster together mainly due to low expressions of the chosen genes.

#### 3.4.2. Expression of Genes Characteristic of Pluripotent Stem Cells

**Figure 5:**
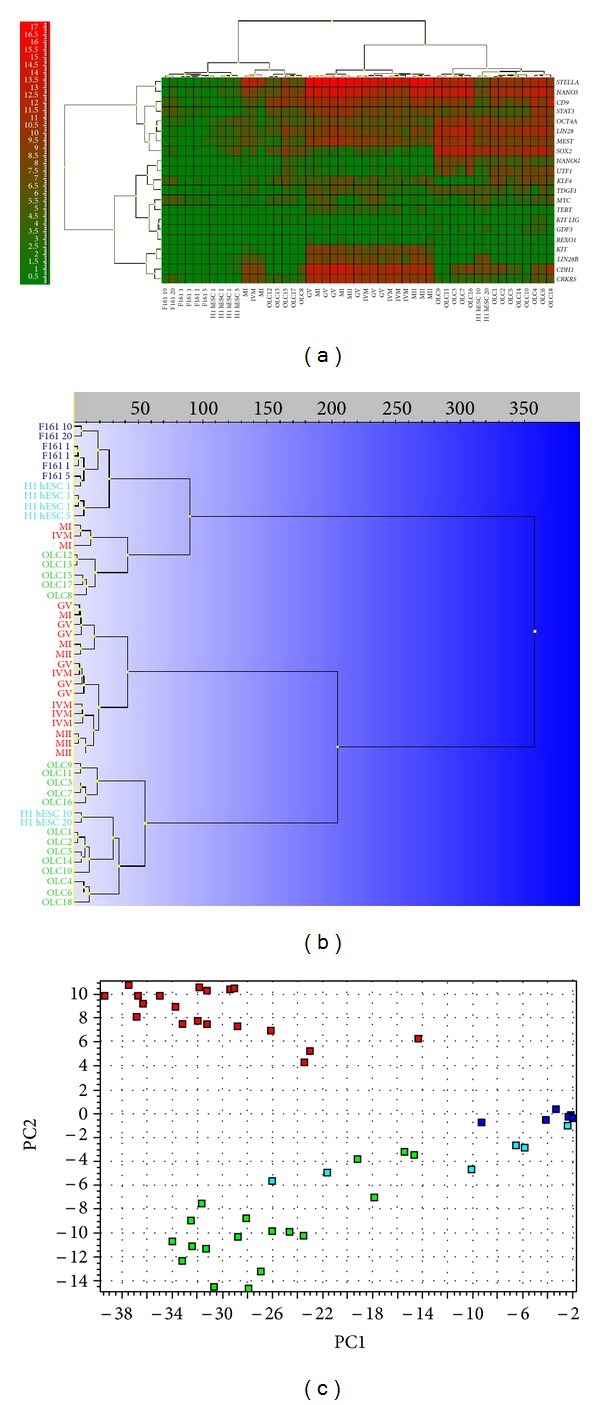
Single-cell gene expression profile of genes related to pluripotency in primitive oocyte-like cells (OLCs) developed *in vitro*. Comparison with human embryonic stem cells (H1 hESCs), nonfertilized oocytes from the *in vitro* fertilization programme (O) and fibroblasts (F161) revealed the expression of several genes related to pluripotency in oocyte-like cells. (a) Heatmap clustering. (b) Hierarchical clustering. (c) Principle component analysis (PC1 versus PC2). Legend of analyzed cells:OLC(1–18): oocyte-like cells developed *in vitro*; H1 hESC: human embryonic stem cells of line H1 (1: one cell, 5: five cells, 10: ten cells, and 20: twenty cells); F161: fibroblasts of F161 line (1: one cell, 5: five cells, 10: ten cells, and 20: twenty cells); MII: mature, metaphase II oocytes; IVM: immature, *in vitro* matured oocytes; MI: immature, metaphase I oocytes; GV: immature, germinal vesicle oocytes from the *in vitro* fertilization programme. Cell group colours: red: oocytes from the *in vitro* fertilization programme, green: oocyte-like cells, developed *in vitro*, aquamarine: hESCs, and dark blue: fibroblasts.

Oocyte-like cells developed *in vitro* expressed 17 out of the 21 analyzed genes characteristic of pluripotent stem cells (Figure 5(a)). Primitive oocyte-like cells expressed *KIT*-*LIG*, *OCT4A*, *NANOG*, *MYC*, *KLF4*, *SOX2*, *UTF1*, *TDGF1*, *CD9*, *LIN28*, *NANOS*, *CDH1*, *STAT3*, *MEST*, *CRKRS*, *STELLA*, and *GDF3* and did not express *KIT*, *LIN28B*, *TERT*, and *REX1*. Most of the 13 cells clustered together with the groups of 10 and 20 hESCs, as revealed by hierarchical clustering (dendrogram) (Figure 5(b)) and PCA (Figure 5(c)). Only five cells clustered with three outstanding oocytes (Figure 5).

**Figure 6:**
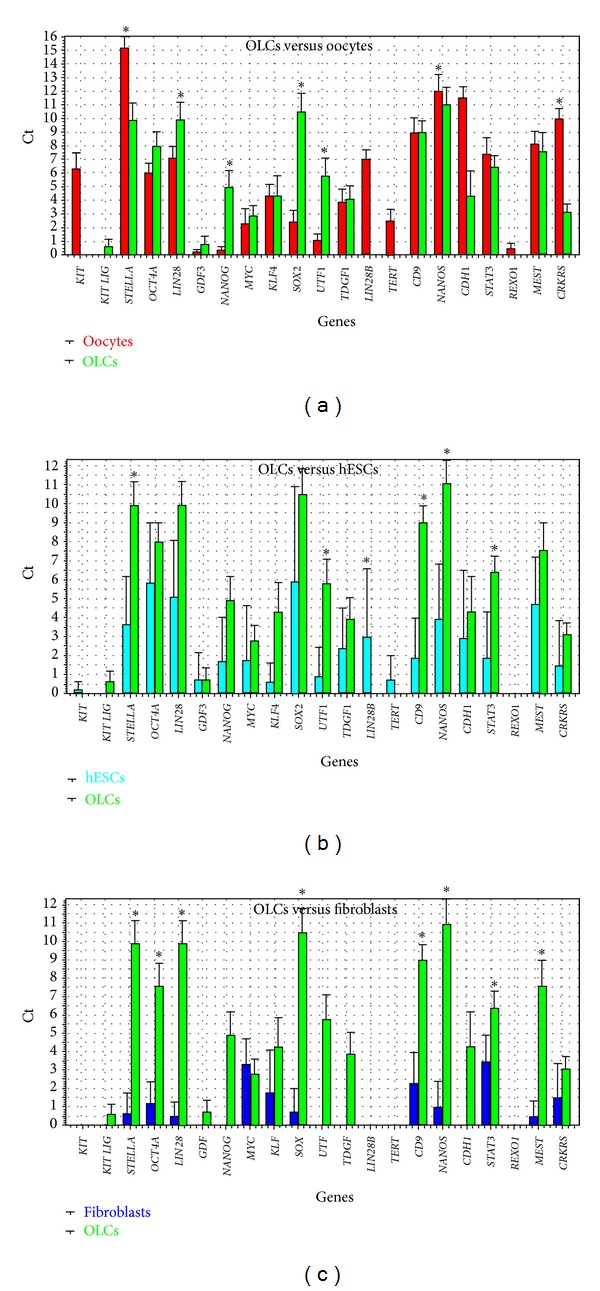
Descriptive statistics of the expression of genes (Ct values), characteristic of pluripotent stem cells in primitive oocyte-like cells (OLCs), developed *in vitro*. (a) Comparison with oocytes. (b) Comparison with hESCs. (c) Comparison with fibroblasts. Cell group colours: green: oocyte-like cells, developed *in vitro*, red: oocytes from the *in vitro* fertilization programme, and dark blue: fibroblasts. Legend: *Statistically significant difference at *P* < 0.00244 (Dunn-Bonferroni correction), as revealed using the two-tailed Mann-Whitney test.

Primitive oocyte-like cells developed *in vitro* expressed *LIN28*, *NANOG*, *SOX-2*, and *UTF1* to significantly a greater extent and *STELLA*, *CDH1*, and *CRKRS* to significantly lesser extent than oocytes, as revealed by two-tailed Mann-Whitney test (Figure 6(a)). To keep the overall risk of false positive at 5%, a *threshold* value of *P* < 0.00244 was used to indicate significance. The *P* values are presented in Table 3. *KIT-LIG* was the only gene not expressed in the oocytes. Primitive oocyte-like cells developed *in vitro* expressed *CD9*, *NANOS, STELLA*, *STAT3*, *UTF1*, and *LIN28 *to significantly greater extents than the hESCs (Figure 6(b)). hESCs did not express *KIT-LIG *and* REX1*. Different from the primitive oocyte-like cells and hESCs, fibroblasts did not express *KIT*, *KIT-LIG*, *GDF3*, *NANOG*, *UTF1*, *TDGF1*, *LIN28B*, *TERT*, *CDH1*,and *REX1*,which are characteristic of pluripotent stem cells (Figure 6(c)). Further, they did express *NANOS*, *LIN28*, *STELLA*, *CD9, SOX-2*, *OCT4A*, *MEST*,and *STAT3* at a very low level, substantially less than the primitive oocyte-like cells (Figure 6(c); *P* values in Table 3). Comparing all groups of cells statistically significant differences (Table 3) were found in the expression of *KIT*, *STELLA*, *OCT4A*, *LIN28*, *NANOG*, *SOX-2*, *UTF1*, *TDGF1*, *LIN28B*, *TERT*, *CD9*, *NANOS*, *CDH1*, *STAT3*, *MEST*, and* CRKRS* based on the One-Way ANOVA.

#### 3.4.3. Expression of Oocyte-Specific Genes in Comparison with Nonfertilized Oocytes from the *In Vitro* Fertilization Programme

**Figure 7:**
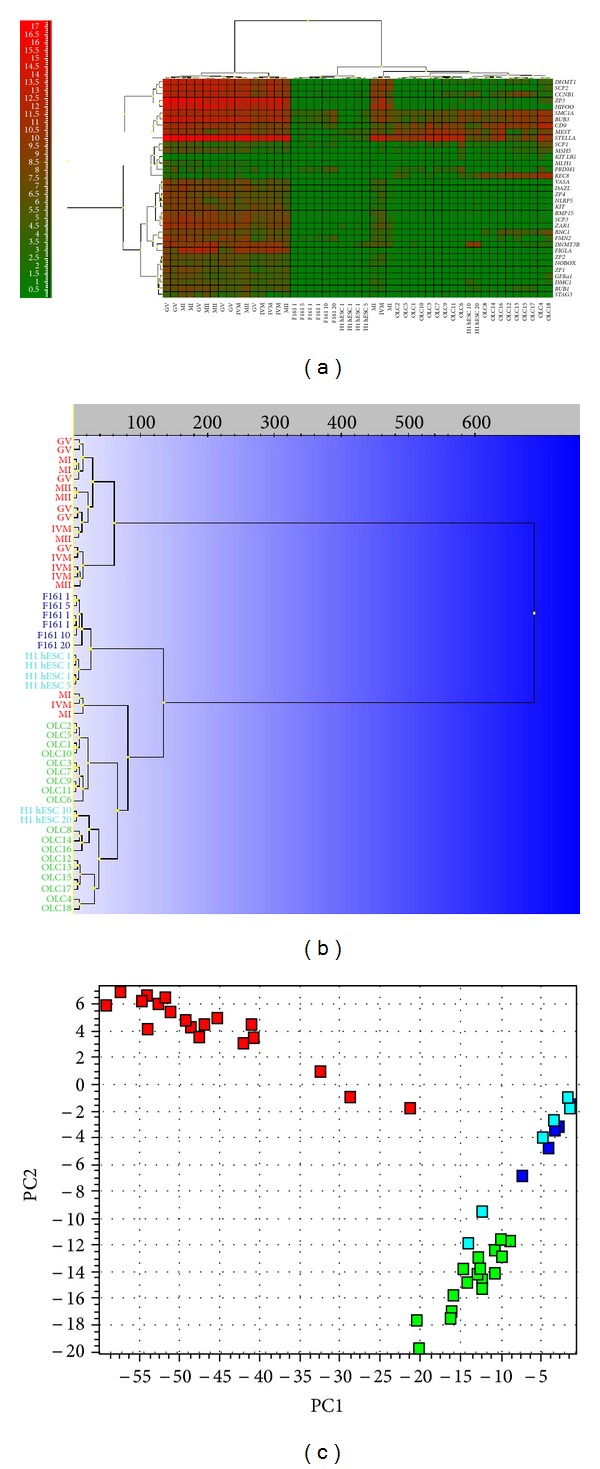
Single-cell gene expression profile of oocyte-specific genes in primitive oocyte-like cells developed *in vitro* (OLCs). Comparison with human embryonic stem cells (H1 hESCs), nonfertilized oocytes from the *in vitro* fertilization programme (O) and fibroblasts (F161) revealed the expression of several oocyte-specific genes in oocyte-like cells. (a) Heatmap clustering. (b) Hierarchical clustering. (c) Principle component analysis (PC1 versus PC2). Legend of analyzed cells:OLC(1–18): oocyte-like cells developed *in vitro*; O: oocytes; H1 hESC: human embryonic stem cells of line H1 (1: one cell, 5: five cells, 10: ten cells, and 20: twenty cells); F161: fibroblasts of F161 line.

Primitive oocyte-like cells developed *in vitro *expressed 22 out of the 34 analyzed oocyte-specific genes (Figure 7(a)): *GFRAa1*, *KIT-LIG*, *DNMT3B*, *DNMT1*, *ZP3*, *SCP1*, *SCP2*, *SCP3*, *CCNB1*, *FMN2*, *HIFOO*, *DMC1*, *BUB1*, *BUB3*, *STELLA*, *STAG3*, *SMC1A*, *BNC1*, *REC8*, *MSH5*, *PRDM1/BLIMP1*, and *CD9 *and did not express the twelve important oocyte- (germline-) specific genes: *VASA*, *DAZL*, *KIT*, *BMP15*, *ZP1*, *ZP2*, *ZP4*, *NOBOX*, *NLRP5*, *MLH1*, *ZAR1*, and *FIGLA*. As revealed by hierarchical clustering (Figure 7(b)) and PCA (Figure 7(c)), the oocytes form an independent cluster evidencing a distinct expression profile with the exception of two immature MI oocytes and one IVM oocyte. Positive control oocytes from the *in vitro* fertilization programme expressed all the oocyte-specific genes except *KIT-LIG*. They expressed *BNC1* at a significantly higher level than *in vitro* developed oocyte-like cells (Table 3) according to the Mann-Whitney test (two-tailed). To keep the risk of false positive at 5%, a *threshold* value of *P* < 0.00151 was used. At this very conservative *threshold* differential expression of other genes was not significant.

**Figure 8:**
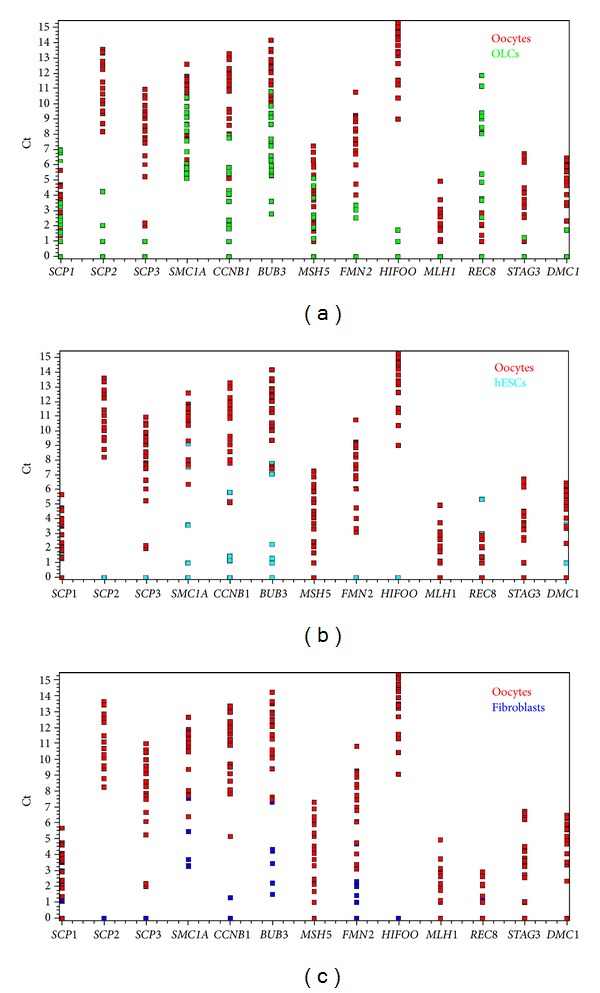
Expression of meiosis-related genes in oocyte-like cells developed *in vitro*. Primitive oocyte-like cells expressed the most, 12 out of 13 meiosis-related genes. In oocyte-like cells gene expressions fitted with oocyte gene expressions while in hESCs and fibroblasts they did not. Expression of meiosis-related genes (Ct values) presented in 2D plots: (a) oocytes (red) and oocyte-like cells (green); (b) oocytes (red) and hESCs (aquamarine); (c) oocytes (red) and fibroblasts (dark blue).

Focusing on 13 oocyte-specific genes that are directly or indirectly involved in meiosis (*SMC1A*, *SCP1*, *SCP2*, *SCP3*, *CCNB1*, *BUB3*, *MSH5*, *FMN2*, *HIFOO*, *MLH1*, *REC8*, *STAG3*, and *DMC1*) we find that the primitive oocyte-like cells developed *in vitro* expressed twelve out of the 13 genes. Only the *MLH1* gene was not expressed (Figure 7(a)). In the primitive oocyte-like cells the meiotic gene *MSH5* was expressed at a significantly lower level than in the nonfertilized oocytes from the *in vitro* fertilization programme, while *REC8 *was expressed at a significantly higher level (two-tailed Mann-Whitney test, significance *threshold* *P* < 0.00394; *P* values presented in Table 3). Differential expression of other genes was not statistically significant based on this conservative *threshold*. The gene expression levels (Ct values) of the primitive oocyte-like cells and of the oocytes matched very well, particularly for *SCP1*, *SMC1A*, and *MSH5*, whereas for the hESCs and the fibroblasts they did not (Figure 8). Although the primitive oocyte-like cells and the oocytes separate two distinct groups of cells based on the overall gene expression profile, as revealed by hierarchical clustering (Figure 7(b)), there are substantial similarities. The hESCs and the fibroblasts express meiotic genes at very low level. Seven genes (*SCP2*, *SCP3*, *MSH5*, *FMN2*, *HIFOO*, *MLH*, and *STAG3*) were not expressed at all in the hESCs, and another set of seven meiotic genes (*SCP2*, *SCP3*, *MSH5*, *HIFOO*, *MLH1*, *STAG3*, and* DMC1*) was not expressed in the fibroblasts (Figure 7(a)).

### 3.5. DAPI Staining the Cell Nuclei

**Figure 9:**
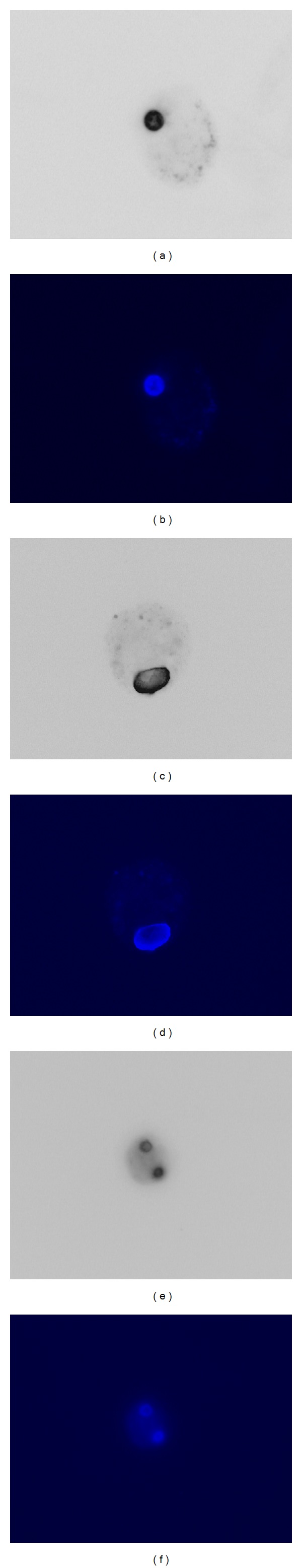
Nuclei of *in vitro* developed primitive oocyte-like cells monitored after DAPI staining. DAPI staining confirmed mononuclear and binuclear cells: ((a)–(d)) two normal mononuclear primitive oocyte-like cells. ((e), (f)) One abnormal primitive oocyte-like cell with two nuclei. (Light and fluorescence microscope, magnification 400x.)

Sixty-eight oocyte-like cells developed *in vitro* and collected from all patients were monitored after DAPI staining: 67 (98.5%) cells were normal, mononuclear, and one cell (1.5%) had two nuclei (Figure 9).

## 4. Discussion

This study confirms the existence of cells expressing several markers of pluripotency—putative stem cells—in the OSE layer of women with POF. In this study the putative ovarian stem cells were scraped from the ovarian surface epithelium of women with nonfunctional ovaries and cultured *in vitro* in the presence of follicular fluid from the *in vitro* fertilization programme. The added follicular fluid triggered development of stem cells into primitive oocyte-like cells, which expressed some markers of pluripotency and oocytes.

Follicular fluid retrieved at the ultrasound-guided oocyte aspiration in the *in vitro* fertilization programme contains several substances important for oocyte growth and maturation including high levels of hormones [30]: estrogens, progesterone and FSH, androgens, proteins [31], and amino acids [31], a high concentration of lipids including free cholesterol and meiosis-activating sterol (FF-MAS) [32, 33], and growth factors [34]. Many of these substances are important for oocyte growth and maturation.

The culture medium with added follicular fluid used in this study was characterized by very high concentrations of estradiol and progesterone. The high concentrations of these reproductive hormones in follicular fluid were normal and resulted from the ovarian hormonal stimulation protocol, which was routinely used in a patient to retrieve oocytes for *in vitro* fertilization. The OSE cell cultures including oocyte-like cells depleted/converted these hormones from the culture medium. It has already been found that follicular fluid can induce OSE cell proliferation, but estradiol and progesterone did not induce the cell proliferation; it was concluded that follicular fluid directly stimulates OSE cell proliferation by nonsteroidal mitogens [35]. On the other hand several studies confirmed that estradiol has a positive impact on the human oocyte growth and maturation *in vivo* and *in vitro*. It has already been published that oocyte-like cell development *in vitro* can be triggered by weak estrogenic activity of phenol red in the cell culture medium [18, 25]. The estradiol can directly influence the quality of maturing human oocytes by steroid action on the cell surface via Ca^2+^ as a second messenger and contribute to the oocyte potential for fertilization and early postfertilization development [36]. In the *in vitro* fertilization programme estradiol and testosterone levels in follicular fluid may be used as predictive parameters of oocyte maturity [37]. In the follicular phase of menstrual cycle, follicles which contained a healthy but not degenerative oocyte had a significantly higher level of estradiol in the follicular fluid [38]. The oocytes which gave rise to successful pregnancies were obtained from follicles which contained greater concentrations of estradiol than did oocytes from which pregnancy did not result [39]. Moreover, transient estradiol supplementation improved the oocyte *in vitro* maturation rate and subsequent developmental competence in porcine [40] and white-tailed deer oocytes [41]. Similarly, estradiol and progesterone improved *in vitro* cytoplasmic maturation and developmental competence of oocytes from unstimulated prepubertal and adult rhesus monkeys [42]. Based on all this knowledge, it is not excluded that estradiol alone or in combination with other substances from the follicular fluid was involved in *in vitro* development of primitive oocyte-like cells in this study.

The majority of primitive oocyte-like cells developed *in vitro* were normally mononuclear and expressed several genes characteristic of pluripotent stem cells and of oocytes based on single-cell gene expression profiling. They express most of the genes of pluripotency tested and several oocyte-specific genes, including genes related to epigenetic regulation (e.g., *DNMT1*, *DNMT3B*), zona pellucida structure (*ZP3*), and late meiotic genes (e.g., *SCP1, SCP2,* and *SCP3*). Although these genes were expressed at a significantly lower extent than in nonfertilized oocytes from the *in vitro* fertilization programme, there was a subgroup of immature or *in vitro* matured oocytes that clustered together with oocyte-like cells and not with other oocytes based on their gene expression profiles.

Although primitive oocyte-like cells less expressed a majority of the analyzed genes involved in meiosis (*SCP1*, *SCP2*, *SCP3*, *SMC1A*, *CCNB1*, *BUB3*, *MSH5*, *FMN2*, *HIFOO*, *MLH1*, *REC8*, *STAG3*, and *DMC1*), they still expressed *REC8* at a significantly higher level than the oocytes. *REC8* plays an important role in sister chromatid cohesion during meiosis [43]. In spite of that, oocyte-like cells did not express some important oocyte-specific genes, such as *VASA, DAZL*, and *FIGLA*, and therefore exhibited the characteristics of pluripotent stem cells more than of competent oocytes. As shown in the model of human and mouse ESCs, the expression of germ cell-specific genes (including *VASA*) in oocyte-like cells developed *in vitro* can be regulated by different culture conditions [7, 10], for example, by the addition of retinoic acid into the culture medium that regulates germ cell differentiation through a Smad-dependent pathway [44]; therefore the possibilities of *in vitro* “oogenesis” in this study were far from being exhausted. As previously proposed, oocyte-like cells developed *in vitro* could also be matured *in vivo* by transplantation into other organs, tissues, or ovarian biopsies [5, 23]; it may be possible to autotransplant the oocyte-like cells to *in vivo* direct their growth, maturity, and function or perhaps to “awaken” the ovaries in women with severe ovarian infertility, if sure that teratoma would not form.

The results of this study are in accordance with findings of White et al. [23], who published the existence of rare mitotically active cells—stem cells—with a gene expression profile that is consistent with primitive germ cells in ovarian cortical tissue of women in reproductive period of age. These cells were able to generate oocyte-like cells *in vitro*.

The results of our study show that the adult OSE in women with nonfunctional ovaries may be an important source of putative stem cells.

## 5. Conclusions

The addition of donated follicular fluid, rich in substances for oocyte growth and maturation, to the culture medium triggers development of putative stem cells from the ovarian surface epithelium of women with nonfunctional ovaries into the direction of primitive oocyte-like cells that express several genes related to pluripotency and oocytes, but are more “stem cells” than real “oocytes” at present.

## Figures and Tables

**Table 1:** Clinical data of all five patients with POF included into this study, which were documented at the Department of Obstetrics and Gynecology, University Medical Centre Ljubljana. All patients had increased serum levels of gonadotropins FSH (normal: <11.3 IU/L) and LH (normal: <11.6 IU/L) and thin endometrium.

Clinical data	Patients				
	P1 (R.A.)	P2 (G.K.)	P3 (M.S.)	P4 (G.A.)	P5 (P.R.A.)
Age (years)	39	40	21	31	39
FSH (IU/L)	89.1	65.8	11.8	162.0	67.8
LH (IU/L)	23.3	23.4	10.1	59.0	37.8
Prolactin (mg/L)	12	4.6	16.4	9.3	/
Estradiol (nmol/L)	<0.073	0.08	0.2	/	/
Inhibin B (ng/L)	<10	<10	52.4	>10	/
S-AMH (mg/L)	0.00	0.00	0.89	0.00	/
Karyotype	Abnormal (mosaic 45X, 47XXX, 48XXXX, 46XX)	Normal (no FMR1-fragile X mutation)	Normal (no FMR1-fragile X mutation)	Normal (no FMR1-fragile X mutation)	Normal (no FMR1-fragile X mutation)
Ovarian cortex histology	Inclusion cysts, corpora albicantia, focal ovarian surface epithelium, no follicles or oocytes	Inclusion cysts, corpora albicantia, simple or stratified ovarian surface epithelium, no follicles or oocytes	Simple columnar ovarian surface epithelium, several primordial follicles	Simple cuboidal ovarian surface epithelium, corpus luteum in regression, no follicles or oocytes	Simple cuboidal ovarian surface epithelium, no follicles or oocytes
Antiovarian antibodies	No	No	Yes	Yes	No
Premature ovarian failure (POF)	Secondary (previous birth of a child), irregularities of menstrual cycles, thin endometrium	Primary (no children), irregular menstrual cycles, thin endometrium	Primary (no children), irregular menstrual cycles, thin endometrium	Primary (no children), amenorrhea, small left ovary, thin endometrium	Secondary (pregnancy ended in spontaneous abortion), amenorrhea, thin endometrium

**Table 2:** The mean concentrations of reproductive hormones in samples of culture medium (DMEM/F-12) with added follicular fluid (FF). Retrieved from two different ovarian cell cultures (OSE) with developing oocyte-like cells after 15–17 days of culturing in comparison with the same culture medium with added follicular fluid (without OSE cell culture) and the same culture medium (without added follicular fluid and OSE cell culture).

	Concentrations of hormones in culture medium			
	Estradiol* (nmol/L)	Progesterone** (nmol/L)	Androstenedione (nmol/L)	Testosterone (nmol/L)
DMEM/F-12 + FF + OSE (min.−max.)	175.5 (149.0–202.0)	1473.5 (3999.0–1052.0)	81.4 (82.5–80.3)	2.55 (2.4–2.7)
DMEM/F-12+ FF (min.−max.)	310.5 (377.0–244.0)	4783.5 (5528.0–4039.0)	91.4 (90.1–92.8)	4.15 (3.9–4.4)
DMEM/F-12 (min.−max.)	0.73 (0.66–0.81)	<0.64	0.015 (0.01-0.02)	1.1 (1.0–1.2)

*Reference serum values in women. Follicular phase: 0–0.59 nmol/L; follicular phase, day 2-3: 0–0.31 nmol/L; at ovulation: 0.12–1.47 nmol/L; luteal phase: 0.1–0.9 nmol/L; nontreated postmenopausal: 0–0.11 nmol/L; treated postmenopausal: 0–0.34 nmol/L; at oral contraception: 0–0.37 nmol/L. Reference serum value in men: 0–0.21 nmol/L.

**Reference serum values in women. Follicular phase: up to 3.6 nmol/L; follicular phase, middle: up to 3.1 nmol/L; at ovulation: 1.5–5.5 nmol/L; luteal phase: 3.0–68 nmol/L; luteal phase, middle: 19–76 nmol/L; postmenopausal: up to 3.2 nmol/L; at oral contraception: 1.1–2.9 nmol/L; pregnancy, first quarter: 29.6–106 nmol/L; pregnancy, second quarter: 93.8–159 nmol/L; pregnancy, third quarter: 264–509 nmol/L. Reference serum value in men: 0.86–2.9 nmol/L.

**Table 3:** *P* values retrieved by statistical analyses of single-cell gene expressions in analyzed groups of cells.

Statistical test	Compared cells	*P* value
Genes of pluripotency		

Two-tailed Mann-Whitney test	OLCs versus oocytes	*LIN28 * (*P* = 0.00078), *STELLA* (*P* = 1.0766 × 10^−6^), *NANOG* (*P* = 8.5203 × 10^−6^), *SOX-2 *(*P* = 2.2075 × 10^−7^), *UTF1* (*P* = 1.1288 × 10^−5^), *CDH1* (*P* = 1.0766 × 10^−6^), *CRKRS* (*P* = 2.2075 × 10^−7^)

Two-tailed Mann-Whitney test	OLCs versus hESCs	*CD9* (*P* = 0.00036), *NANOS* (*P* = 0.00097), *STELLA* (*P* = 0.00154), *STAT3* (*P* = 0.00154), *UTF1* (*P* = 0.00154), *LIN28* (*P* = 0.00242)

Two-tailed Mann-Whitney test	OLCs versus fibroblasts	*NANOS* (*P* = 0.00036), *LIN28* (*P* = 0.00036), *STELLA* (*P* = 0.00036), *CD9* (*P* = 0.00036), *SOX-2 *(*P* = 0.00036), *OCT4A* (*P* = 0.00036), *MEST* (*P* = 0.00060), *STAT3* (*P* = 0.00242)

One-Way ANOVA	All types of cells	*KIT* (*P *≤ 1 × 10^−8^), *STELLA* (*P* ≤ 1 × 10^−8^), *OCT4A* (*P* = 6.86 × 10^−6^), *LIN28* (*P* ≤ 1 × 10^−8^), *NANOG* (*P* = 1.3 × 10^−7^), *SOX-2* (*P* ≤ 1 × 10^−8^), *UTF1* (*P* = 1 × 10^−8^), *TDGF1* (*P* = 0.00167408), *LIN28B* (*P* ≤ 1 × 10^−8^), *TERT* (*P* ≤ 1 × 10^−8^), *CD9* (*P* ≤ 1 × 10^−8^), *NANOS* (*P* ≤ 1 × 10^−8^), *CDH1* (*P* ≤ 1 × 10^−8^), *STAT3* (*P* = 4.67 × 10^−6^), *MEST* (*P* = 5.7 × 10^−7^), *CRKRS* (*P* ≤ 1 × 10^−8^).

Oocyte-specific genes		

Two-tailed Mann-Whitney test	OLCs versus oocytes	*BNC1* (*P* = 0.00011)

Genes of meiosis		

Two-tailed Mann-Whitney test	OLCs versus oocytes	*MSH5* (*P* = 0.00204), *REC8* (*P* = 0.00166)

Legend: OLCs: oocyte-like cells; hESCs: human embryonic stem cells.
